# Antibody to Aquaporin 4 in the Diagnosis of Neuromyelitis Optica

**DOI:** 10.1371/journal.pmed.0040133

**Published:** 2007-04-17

**Authors:** Friedemann Paul, Sven Jarius, Orhan Aktas, Martin Bluthner, Oliver Bauer, Heribert Appelhans, Diego Franciotta, Roberto Bergamaschi, Edward Littleton, Jacqueline Palace, Hans-Peter Seelig, Reinhard Hohlfeld, Angela Vincent, Frauke Zipp

**Affiliations:** 1 Cecilie-Vogt-Clinic for Molecular Neurology, Charité, University Medicine Berlin, Berlin, Germany; 2 Max-Delbrueck-Center for Molecular Medicine, Berlin, Germany; 3 Institute of Clinical Neuroimmunology, University of Munich, Munich, Germany; 4 Department of Clinical Neurology, University of Oxford, Oxford, United Kingdom; 5 Weatherall Institute of Molecular Medicine, University of Oxford, Oxford, United Kingdom; 6 Institute of Immunology and Molecular Genetics, Karlsruhe, Germany; 7 Istituto di Ricovero e Cura a Carattere Scientifico, Foundation Neurological Institute C. Mondino, University of Pavia, Pavia, Italy; University of Vienna, Austria

## Abstract

**Background:**

Neuromyelitis optica (NMO) is a demyelinating disease of the central nervous system (CNS) of putative autoimmune aetiology. Early discrimination between multiple sclerosis (MS) and NMO is important, as optimum treatment for both diseases may differ considerably. Recently, using indirect immunofluorescence analysis, a new serum autoantibody (NMO-IgG) has been detected in NMO patients. The binding sites of this autoantibody were reported to colocalize with aquaporin 4 (AQP4) water channels. Thus we hypothesized that AQP4 antibodies in fact characterize NMO patients.

**Methods and Findings:**

Based on these observations we cloned human water channel *AQP4,* expressed the protein in a eukaryotic transcription/translation system, and employed the recombinant AQP4 to establish a new radioimmunoprecipitation assay (RIPA). Indeed, application of this RIPA showed that antibodies against AQP4 exist in the majority of patients with NMO (*n* = 37; 21 positive) as well as in patients with isolated longitudinally extensive transverse myelitis (*n* = 6; six positive), corresponding to a sensitivity of 62.8% and a specificity of 98.3%. By contrast, AQP4 antibodies were virtually absent in 291 other participants, which included patients with MS (*n* = 144; four positive), patients with other inflammatory and noninflammatory neurological diseases (*n* = 73; one positive), patients with systemic autoimmune diseases (*n* = 45; 0 positive), and healthy participants (*n* = 29; 0 positive).

**Conclusions:**

In the largest series reported so far to our knowledge, we quantified AQP4 antibodies in patients with NMO versus various other diseases, and showed that the aquaporin 4 water channel is a target antigen in a majority of patients with NMO. The newly developed assay represents a highly specific, observer-independent, and easily reproducible detection method facilitating clinically relevant discrimination between NMO, MS, and other inflammatory diseases.

## Abstract 2

A newly developed method to detect antibodies to the aquaporin 4 water channel can help discriminate between neuromyelitis optica, multiple sclerosis, and other inflammatory diseases.

## Editors' Summary

**Background.:**

Neuromyelitis optica (NMO or Devic syndrome) is a rare disease in which the immune system destroys the myelin (fatty material that insulates nerve fibers so that the body and the brain can communicate using electrical messages) in the optic nerve and spinal cord. Myelin destruction (demyelination) in these parts of the central nervous system (CNS) causes pain and swelling (inflammation) of the optic nerve (optic neuritis) and spinal cord (myelitis). The resultant disruption of communication along these nerves means that patients with NMO experience temporary or permanent blindness in one or both eyes that is preceded or followed by limb weakness or paralysis and loss of bladder and bowel control. These two sets of symptoms can occur many months apart and may happen once during a person's lifetime or recur at intervals. There is no cure for NMO, but corticosteroids or plasmapheresis reduce inflammation during acute attacks and, because NMO is an autoimmune disease (one in which the immune system attacks the body's own tissues instead of foreign organisms), long-term immunosuppression may prevent further attacks.

**Why Was This Study Done?:**

There are many inflammatory/demyelinating diseases of the CNS with clinical symptoms similar to those of NMO. It is particularly hard to distinguish between NMO and multiple sclerosis, an autoimmune disease that involves widespread demyelination. Neurologists need to make a correct diagnosis before starting any treatment and usually use clinical examination and magnetic resonance imaging (to detect sites of inflammation) to help them in this task. Recently, however, a biomarker for NMO was identified. Many patients with NMO make autoantibodies (proteins that recognize a component of a person's own tissues) called NMO-IgGs. These recognize aquaporin 4 (AQP4), a protein that allows water to move through cell membranes. It is not known how often patients with NMO or other demyelinating diseases make antibodies to AQP4, so it is unclear whether testing for these antibodies would help in the diagnosis of NMO. In this study, the researchers have developed a new assay for antibodies to AQP4 and then quantified the antibodies in patients with NMO and other demyelinating diseases.

**What Did the Researchers Do and Find?:**

The researchers made radioactively labeled AQP4 in a test tube, then incubated samples of this with serum (the liquid portion of blood), added small beads coated with protein A (a bacterial protein that binds to antibodies) and allowed the beads to settle. The amount of radioactivity attached to the beads indicates the amount of antibody to AQP4 in the original serum. The researchers used this radioimmunoprecipitation assay to measure antibodies to AQP4 in sera from 37 patients with NMO and from six with another neurological condition, longitudinally extensive transverse myelitis (LETM), which is characterized by large demyelinated lesions across the width of the spinal cord but no optic neuritis; these patients often develop NMO. They also measured antibodies to AQP4 in the sera of nearly 300 other people including patients with multiple sclerosis, other neurological conditions, various autoimmune diseases, and healthy individuals. Nearly two-thirds of the patients with NMO and all those with LETM made antibodies against AQP4; very few of the other study participants made these antibodies. In particular, only four of the 144 patients with multiple sclerosis made AQP4 antibodies.

**What Do These Findings Mean?:**

These findings indicate that testing for antibodies to AQP4 could help neurologists distinguish between NMO and multiple sclerosis and between NMO and other demyelinating diseases of the CNS. In addition, the new radioimmunoprecipitation assay provides a standardized, high-throughput way to quantitatively test for these antibodies, whereas the indirect immune fluorescence assay for measurement of unspecific NMO-IgG is observer-dependent and nonquantitative. Although these findings need to be confirmed in more patients and the assay's reliability demonstrated in different settings, the measurement of antibodies to AQP4 by radioimmunoprecipitation may become a standard part of the differential diagnosis of NMO. Additional research will determine whether AQP4 is the only protein targeted by autoantibodies in NMO and whether this targeting is a critical part of the disease process.

**Additional Information.:**

Please access these Web sites via the online version of this summary at http://dx.doi.org/10.1371/journal.pmed.0040133.

- US National Institute of Neurological Disorders and Stroke has information for patients who have neuromyelitis optica, transverse myelitis, and multiple sclerosis
- The Transverse Myelitis Association offers information and useful links for patients and their carers about transverse myelitis and neuromyelitis optica (in several languages, including English and Spanish)
- Mayo Clinic information for patients on Devic's syndrome
- Medline Plus encyclopedia pages discuss autoimmune disorders  (in English and Spanish)
- A brief overview of aquaporins is available from the University of Miami
- The American MS Society has information on MS

## Introduction

Neuromyelitis optica (NMO, Devic syndrome) is a chronic inflammatory demyelinating disease of the central nervous system (CNS), mainly affecting the optic nerves and the spinal cord [1–4]. It is still a matter of debate whether NMO represents a disease entity in itself or whether it is a subform of multiple sclerosis (MS). Like MS, NMO follows a relapsing–remitting course in the majority of cases, but it usually leads to more severe disability with impairment of functional vision and/or loss of ambulation. Early differentiation of NMO from MS and other inflammatory and demyelinating diseases of the CNS—such as vasculitis, neuroborreliosis, paraneoplastic neurological syndromes, or vitamin B12 deficiency—is highly desirable, as treatment options and prognoses differ widely. However, such differentiation may be difficult or even impossible owing to overlap in clinical presentation and cerebrospinal fluid (CSF) and magnetic resonance imaging (MRI) findings [5].

A serum autoantibody (called NMO-IgG) binding to CNS microvessels, pia, subpia, and Virchow-Robin spaces was recently identified in patients with NMO, using indirect immunofluorescence [6]. The binding sites of this autoantibody were then reported to colocalize with aquaporin 4 (AQP4) water channels in mouse tissue of the brain, stomach and kidney, generating the hypothesis of NMO as an autoimmune channelopathy [7]. However, to date there has been no systematic assessment of the occurrence and frequency of AQP4 antibodies in NMO and other demyelinating diseases. It is therefore still unclear whether antibodies to AQP4 are specific for NMO and whether testing for these antibodies may be useful for its diagnosis and its distinction from diseases with similar clinical and neuroradiological patterns, such as MS and other autoimmune diseases with involvement of the CNS. The aim of our study was to assess the frequency and specificity of antibodies to AQP4 in patients with NMO, as well as relevant differential diagnoses, by means of a newly developed radioimmunoprecipitation assay (RIPA).

## Methods

### Patients

NMO patients meeting the diagnostic criteria proposed by Wingerchuk et al. [1] and patients with longitudinally extensive transverse myelitis (LETM) were recruited from several neurology centers in Germany, Italy, and Great Britain. Only patients for whom complete clinical and paraclinical data were available (i.e., clinical course, laboratory and CSF findings, MRI scans of the brain and spinal cord) were eligible for participation in the study. Based upon these selection criteria, 37 patients with NMO were included. Spinal cord lesions extending over three or more vertebral segments in MRI were present in all NMO patients, thus also fulfilling the recently published revised criteria [8]. Another six patients had isolated LETM (monophasic in three, relapsing in three), with spinal lesions extending over three or more segments, but without optic neuritis, and were thus considered to be at “high risk of NMO” according to previous proposals [9]. In addition, tests were carried out on serum samples from 217 patients with miscellaneous inflammatory and noninflammatory neurological disorders (among them 144 patients with MS fulfilling the panel criteria [10]), 45 patients with various rheumatological autoimmune diseases, and 29 healthy participants included as controls (for diagnostic categories see Table 1). Control participants were recruited from the Cecilie-Vogt-Clinic for Molecular Neurology in Berlin, Germany, the Clinic of Rheumatology in Berlin, Germany, and the Institute of Clinical Neuroimmunology in Munich, Germany. Serum testing in patients was approved by the ethics committees of the participating institutions.

**Table 1:**
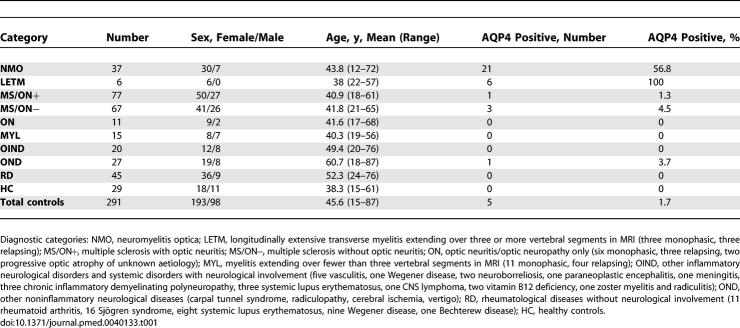
Study Population and Proportion of AQP4-Positive Individuals in Ten Diagnostic Categories

### RIPA

Following PCR-mediated amplification with suitable primer pairs, the cDNA-sequence of human aquaporin 4 (full-length cDNA, amino acids 1–323) was cloned into a pCITE4a vector (Novagen/Merck Biosciences, http://www.merckbiosciences.com). In vitro transcription/translation (IvTT) reactions were performed with the cDNA constructs using a commercial system according to the manufacturer's instructions (Promega, http://www.promega.com). Briefly, 25 μl of rabbit reticulocyte lysate, 2 μl of reaction buffer, 1 μl of amino acid mixture (without methionine), 1 μl of T7 polymerase, 3.5 μl of Redivue L-^35^S-methionine (GE Healthcare, http://www4.gelifesciences.com), 1 μl of RNasin (Cambrex BioScience, http://www.cambrex.com), and 1 μg of template DNA were pipetted into a nuclease-free 1.5 ml reaction tube. Nuclease-free water was added to a final volume of 50 μl. After 90 min incubation at 30 °C, unincorporated ^35^S-methionine was removed by gel filtration on a sephadex G-50 MicroSpin column (GE Healthcare). The percentage of incorporated ^35^S-methionine was calculated using an LS1801 liquid scintillation counter (Beckman, http://www.beckmancoulter.com). Quality and concentration of labeled recombinant protein were analyzed by SDS-PAGE and autoradiography.

Recombinant ^35^S-methionine-AQP4 was diluted in 20 mM Tris-HCl (pH 7.5), 150 mM NaCl, 0.15% Tween-20, 100,000 KIE Trasylol, 10 mM benzamidine, and 0.1% bovine serum albumin (working dilution) to yield an activity of 40,000 cpm in 50 μl. Serum sample aliquots of 5 μl each were added to a 96-well plate, and 50 μl of ^35^S-methionine-AQP4 working dilution were added to each well. Following incubation for 3 h at 4 °C on a shaker, 20 μl protein A Fractogel beads (Merck) were added to each well. After incubation for 1 h at 4 °C the reaction mixtures were transferred to a 0.65 μm Durapore 96-well filter plate (Millipore, http://www.millipore.com). Unbound antigen was removed by vacuum filtration and washing of the filter plates (30 times) with 100 μl of 20 mM Tris-HCl, 150 mM NaCl, and 0.05% Tween-20 each time. After drying the plates overnight, 20 μl liquid scintillation mix MicroScintO (PerkinElmer, http://www.perkinelmer.com) were added to each well and the plates were counted using a Topcount NXT 96-well liquid scintillation counter (PerkinElmer). All determinations were performed in duplicate. A rabbit control antibody (rbAQP4) was prepared, using standard immunization techniques, by immunizing a New Zealand white rabbit with a human AQP4 peptide cocktail.

Mean cpm of five negative controls (healthy blood donors) were used to calculate the antibody ratio (Ab-ratio) according to a formula derived from Frey et al. [11]: Ab-ratio = sample cpm / [mean negative cpm (five negative controls) + f × SD] × 10. Factor f was 3.041, calculated on the basis of a confidence interval of 97.5%. Cutoff was also calculated by means of the equation suggested by Frey et al. [11]. All results with an Ab-ratio >11 were considered positive. All samples were coded with an alphanumerical ID and were analyzed blind. Testing of the rabbit control antibody yielded an Ab-ratio of 133.2.

### Statistics

Data processing (descriptive statistics: mean, range, and standard deviation [SD]) and statistical analyses (antibody positivity between groups: Fisher exact test; antibody ratios between groups: Mann-Whitney test) were performed using SPSS version 12.0 (http://www.spss.com).

## Results

### Establishment of a RIPA Based on Recombinant Human AQP4

**Figure 1:**
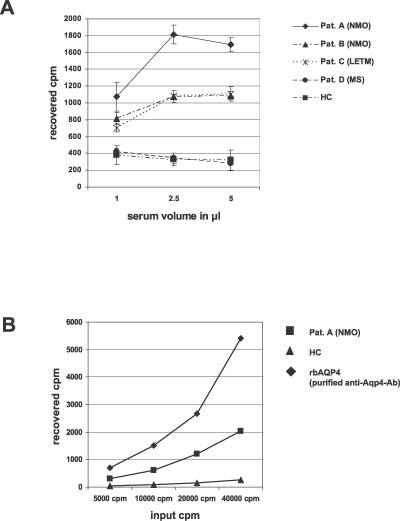
Characteristics of the Radioimmunoprecipitation Assay (A) Titration curve to assess the optimal serum volumes. Five samples are plotted: three positive (two NMO, one LETM) and two negative (one MS, one healthy control [HC]). While positive sera bound up to 1,800 cpm of ^35^S-AQP4 with maximum binding at 2.5–5 μl, negative samples showed no more than 300–400 cpm of precipitation at all serum volumes. Values represent mean cpm of five independent measurements (error bars: SD). (B) Immunoprecipitation at different concentrations of ^35^S-AQP4 (input cpm ranging from 5,000 to 40,000) of a positive serum (NMO), a negative serum (HC), and the rabbit serum rbAQP4 (5 μl per sample).

Using a eukaryotic transcription/translation system, we expressed full-length human AQP4 protein in a cell-free system, labeled with ^35^S, and established a RIPA. By performing titrations with several different volumes of AQP4 positive and negative sera, we determined 5 μl to be the suitable serum volume for routine analysis (Figure 1A). We also tested two sera and the rabbit anti-AQP4 antibody (5 μl per sample) at different concentrations of ^35^S-AQP4 and used the highest activity of ^35^S-AQP4 for the subsequent investigations (Figure 1B). Determination of assay variation was performed for both the rabbit serum rbAQP4 and the sera from nine individual patients by screening ten samples from each donor either on one single occasion (intra-assay variation) or on ten different occasions (interassay variation). The mean intra-assay coefficient of variation was 4.0% (SD 1.3%) and the mean interassay coefficient of variation was 20.7% (SD 2.4%) across the range of the assay. We also measured AQP4 antibodies longitudinally in an LETM patient over a period of 1 y, recording persistent antibody ratios between 23 and 28 (three measurements) during the first 6 mo, and a subsequent gradual decline to 16 under immunosuppression during the following 6 mo while the total amount of IgG remained unchanged (unpublished data).

### Frequency of AQP4 Antibodies in Patient Populations

**Figure 2:**
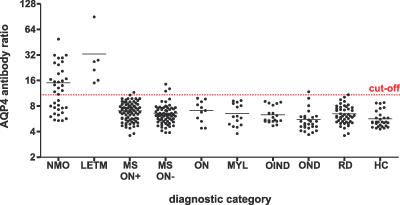
Antibody Ratios Found in Patients with NMO and in Patients with Relevant Differential Diagnoses or Healthy Controls Each individual value represents the result from a single serum sample tested twice with the AQP4 RIPA. Black horizontal bars represent mean. The dashed horizontal line indicates the cutoff at an antibody ratio of 11. Diagnostic categories: NMO, neuromyelitis optica; LETM longitudinally extensive transverse myelitis; MS/ON+, multiple sclerosis with optic neuritis; MS/ON−, multiple sclerosis without optic neuritis; ON, optic neuritis; MYL, myelitis extending over fewer than three vertebral segments on MRI; OIND, other inflammatory neurological disorders and systemic disorders with neurological involvement (e.g., neuroborreliosis, chronic inflammatory demyelinating polyneuropathy; for exact classification see legend for Table 1); OND, other noninflammatory neurological diseases (e.g., carpal tunnel syndrome); RD, rheumatological diseases without neurological involvement (for exact classification see legend for Table 1); HC, healthy controls.

Next we tested sera from the different diagnostic categories. AQP4 antibodies were present (i.e., Ab-ratio > 11) in 21/37 patients with NMO and in 6/6 of those with LETM, but in only 5/291 control samples, including 4/144 with MS and 1/73 with other neurological diseases, 0/45 with rheumatological diseases, and 0/29 healthy individuals (Fisher exact test, Chi-square values ranging from 11.099 [NMO group versus optic neuritis/optic neuropathy group, *p* = 0.001] to 49.353 [NMO versus MS with optic neuritis group], *p* < 0.001 for all other tests) (Figure 2; Table 1). All four MS patients who tested positive were female, had Ab-ratios <15 and an onset age >30 y, and had mainly clinical symptoms related to spinal cord lesions, yet they did not meet the 1999 Wingerchuk criteria for NMO. All four patients had oligoclonal bands and none had spinal cord lesions extending over three or more vertebral segments. The patient from the group of individuals with other neurological diseases who tested positive in the RIPA had carpal tunnel syndrome and showed an Ab-ratio just above the cutoff. The results correspond to a sensitivity of 56.8% and a specificity of 98.3% for the test in NMO, and to 62.8% and 98.3%, respectively, if NMO and LETM patients are considered together. The positive and negative likelihood ratio reveal 33.03 and 0.44 for NMO, and 36.54 and 0.38 for NMO and LETM patients together. Mean antibody ratios in the NMO group were significantly higher than in the other groups (Mann-Whitney test, z-values ranging from −2.736 [NMO group versus optic neuritis/optic neuropathy group, *p* = 0.006] to −5.745 [NMO versus healthy controls], *p* < 0.001 for testing against all other groups). Different levels of antibody binding may indicate the existence of different affinity antibodies or different epitopes recognized in these patients. All positive samples were negative when tested against three control antigens: pemphigus vulgaris-associated autoantigen desmoglein 3; M. Addison- and autoimmune polyendocrine syndrome-associated steroid 21-hydroxylase; and the myositis-associated autoantigen Ku 80, expressed in the same vector system (unpublished data).

## Discussion

This study is, to our knowledge, the first to report on the frequency of autoantibodies recognizing the AQP4 water channel in patients with NMO and to evaluate its specificity when compared to an adequate control group of different patient populations and healthy individuals. Based on the seminal observations of Lennon and colleagues suggesting AQP4 as the target antigen in NMO [7], we cloned and expressed human AQP4 to establish a quantitative radioimmunoprecipitation assay. This novel approach allows standardized high-throughput analysis. In our series of patients with NMO and related disorders, we show that antibodies to the AQP4 water channel are present in 63% of patients with NMO or at high risk of the disease, but are virtually absent in patients with MS and other inflammatory and noninflammatory neurological diseases (5/217), as well as in patients with rheumatological diseases and in healthy controls (0/74). A methodological limitation here is the moderate interassay variation coefficient in this type of assay, although this can be overcome because of the very low intra-assay variation coefficient and the fact that a high number of samples can be measured simultaneously.

Recently, the general presence of a brain-specific autoantibody, designated NMO-IgG, was described in NMO and determined in patients by indirect immunofluorescence [6]. This method of testing for NMO-IgG entails laborious and cost-intensive preabsorption of patient sera, employing blocking procedures in order to avoid unspecific binding and false-positive results. Our detection assay for the specific antibody AQP4 provides observer-independent. quantitative data, and is therefore easily reproducible. It remains to be shown whether all NMO-IgG-positive patients reported also reveal reactivity against the AQP4 water channel.

In our study, antibodies to the AQP4 water channel were sufficiently specific for NMO to allow differentiation from MS and other inflammatory neurological diseases. This finding is of clinical relevance, as diagnosis of NMO based solely on clinical, neuroradiological, and CSF findings may be difficult or impossible. One question that has yet to be answered satisfactorily is whether testing for AQP4 antibodies allows diagnosis of NMO after the first event (i.e., first optic neuritis or myelitis). Weinshenker et al. [9] recently demonstrated that NMO-IgG seropositivity at the initial presentation of LETM predicts relapse of myelitis or development of optic neuritis. All six patients in our series with isolated LETM were positive for antibodies to AQP4, a finding that supports the hypothesis that LETM is an inaugural or limited form of NMO [9]. We did not, however, detect the antibody in patients with less extensive lesions, i.e., myelitis involving fewer than three segments.

Finally, our findings suggest that testing for AQP4 antibodies not only enables a reliable distinction to be made between NMO and MS, but also facilitates differential diagnosis concerning other autoimmune diseases affecting the CNS. This ability to distinguish is particularly important when patients presenting with myelitis have concomitant serological findings suggesting systemic autoimmunity. Positive testing for autoantibodies (ANA, SS-A, SS-B) has been reported in 38%–75% of patients with NMO [12,13], which poses diagnostic challenges to clinicians in many cases. The fact that sera positive for AQP4 antibodies did not show reactivity in testing for the presence of autoantibodies against three unrelated antigens using the same assay system largely rules out nonspecific binding as a cause for anti-AQP4 positivity, and argues for the reliability and specificity of our assay. The negative results in the rheumatological disease group also argue against nonspecific binding of patients' sera to AQP4. Further studies will have to address the question of whether AQP4 is the only target antigen in NMO, and whether antibodies to AQP4 are pathogenic in NMO or a mere epiphenomenon of the disease.

### Accession Numbers

The Swiss-Prot (http://www.expasy.org/sprot) accession number for aquaporin 4 is P55087.

## Abbreviations

CNS: central nervous system
CSF: cerebrospinal fluid
LETM: longitudinally extensive transverse myelitis
MRI: magnetic resonance imaging
MS: multiple sclerosis
NMO: neuromyelitis optica
RIPA: radioimmunoprecipitation assay
SD: standard deviation
